# Polymer Processing: Modeling and Correlations Finalized to Tailoring Plastic Part Morphology and Properties

**DOI:** 10.3390/ma12081217

**Published:** 2019-04-14

**Authors:** Giuseppe Titomanlio, Vito Speranza

**Affiliations:** Department of Industrial Engineering, University of Salerno, via Giovanni Paolo II, 132, 84084 Fisciano, Italy; gtitomanlio@unisa.it

**Keywords:** injection molding, laser-assisted thermal imprinting, extrusion, additive manufacturing, microinjection molding, numerical simulation, morphology, film stretching, composite laminates

## Abstract

The analysis of polymer processing operations requires the description of simultaneous transient momentum and heat transfer down to material solidification. The aim of the analysis is to improve and, hopefully, optimize the final properties that are determined by the final morphology of the part. In this special issue, consisting of 1 review and 11 research articles detailing several polymer processing operations, experimental and numerical analyses have been conducted in order to identify and describe the main relevant phenomena, that affect the product morphologies and properties.

During the transformation of polymeric materials into final usable objects, the polymers (usually viscoelastic fluids) undergo very complex histories of deformation and temperature distributions. The morphology of each element of the final object is determined by deformation and temperature history of the particle which, at the end of a complex evolution, solidified in that position. It is well known that also crystallization kinetics, when active, is deeply influenced by the molecular stretch acquired by effect of the flow. On the other hand, the distribution of final morphology determines the properties of the final object and these can undergo remarkable changes (more than one order of magnitude) by effect of morphology variations.

These observations have been shared for some time within the scientific community, however, only in special cases, clear understanding or description of phenomena taking place along the chain processing-morphology-properties have been identified and reported. On the basis of these considerations the objective of this special issue was the collection of progress or reviews clarifying relationships among processing conditions, as well as the resulting morphology and properties of the final objects. This objective includes both experimental correlations and modelling both in relation to any polymer processing operation and also to any polymer of technological interest.

This special issue includes 11 research articles and 1 review. Nagato et al. investigated the replication of microlens arrays on polymethylmethacrylate (PMMA) films produced by using a laser-assisted thermal imprinting process (LATI) under different processing conditions of pressure and laser power heating mold surface locally [1]. Sun et al. developed a 3D numerical simulation of reactive extrusion processes with the aim of better understanding the effect of operational and geometric parameters on both mixing and reaction processes in the preparation of PP/TiO_2_ nanocomposites [2]. Speranza et al. accurately analyzed morphology via atomic force microscopy (AFM) and discussed, in relation to the operating conditions, the morphologies developed along the cross sections of moldings, obtained by adopting a system able to rapidly change the cavity surface temperature during the process [3]; the processes adopted to obtain those samples were numerically simulated in order to apply a criterion for the achievement of fibrillar morphology based on histories of molecular stretching and mechanical work; the results of the criterion were found to be consistent with the morphology distributions along the cross section of each sample by Liparoti et al. [4]. Li et al. proposed an adaptive optimization method in order to reduce stress and deformation exerted on a polymer stent obtained with the micro-injection molding process [5]. Hashimoto et al. carried on an experimental study on ultra-high molecular weight polyethylene (UHMWPE) films stretched under different operating conditions, namely adopting different temperatures and stretching speeds, and with different operational configurations, by adopting both uniaxial and biaxial stretching modes, in order to evaluate the stretching effect on the film final structure [6]. Zhu et al adopted a Lagrangian approach to obtain numerical results about the mixing mechanism and performance of a novel four-screw extruder [7]. Hamidi et al. investigated processability and properties of silk reinforced composites, obtained with vacuum assisted resin transfer molding (VARTM) [8]. Liparoti et al., analyzed the replication of micro-features and nano-features on PLA molded samples, obtained by injection-molding tests performed with a modulated cavity surface temperature during the process [9]. Hao et al. provided an innovative preparation method of the insulation pressboards adopted in converter transformers; the method is based on the coating of the pressboards with a polytetrafluoroethylene (PTFE) functional film by radio frequency magnetron sputtering; the method was found effective in enhancing both the electrical insulation and the oil insulation of the prepared pressboards [10]. Ruan predicted the effect of flow and temperature on the spherulitical and shish-kebab structures by adopting a simulation model based on a multiscale approach [11]. At last, Gonzalez-Gutierrez et al. prepared a review on the material extrusion additive manufacturing (MEAM) techniques: in particular, they focused the review on the techniques that adopt polymers filled with high contents of ceramic and metallic powders [12]. 
